# Identification of differentially expressed non-coding RNAs in embryonic stem cell neural differentiation

**DOI:** 10.1093/nar/gks922

**Published:** 2012-10-22

**Authors:** Konstantinia Skreka, Simon Schafferer, Irina-Roxanna Nat, Marek Zywicki, Ahmad Salti, Galina Apostolova, Matthias Griehl, Mathieu Rederstorff, Georg Dechant, Alexander Hüttenhofer

*Nucleic Acids Res.* 2012; **40**, 6001–6015. doi:10.1093/nar/gks311

The authors wish to apologize for an error in their article. The correct Sequence Read Archive accession number is SRP010386, not SRP008250.

This correction does not influence the validity of the results and conclusions of this article.

